# Exploring the Impact of Self‐Efficacy on Depression and Anxiety Symptoms in Mild Cognitive Impairment Patients and Their Care Partners: A Dyadic Analysis

**DOI:** 10.1002/alz.085568

**Published:** 2025-01-09

**Authors:** Dianxu Ren, Lisa K Tamres, Jennifer H Lingler

**Affiliations:** ^1^ University of Pittsburgh, Pittsburgh, PA USA; ^2^ University of Pittsburgh Alzheimer’s Disease Research Center (ADRC), Pittsburgh, PA USA

## Abstract

**Background:**

Research on the relationship between self‐efficacy and symptoms of depression and anxiety among individuals with mild cognitive impairment (MCI) has been limited. Furthermore, few studies have explored this relationship within the context of dyadic couples (patient/care partners) rather than focusing solely on individuals. The purpose of this study was to examine the associations between self‐efficacy in patient/care partner couples dealing with mild cognitive impairment and their symptoms of depression and anxiety using a dyadic analysis approach.

**Method:**

We utilized baseline data from a NIH‐funded study for this cross‐sectional analysis. Participants were interviewed regarding self‐efficacy using The Coping Self‐Efficacy Scale, depressive symptoms using the Center for Epidemiological Studies Depression scale, and state anxiety using the Spielberger State‐Trait Anxiety Inventory. We employed the Actor‐Partner Interdependence Model (APIM) to examine the relationships between outcomes (depressive symptoms and state anxiety) and the predictor (self‐efficacy scale) for patients with MCI and their care partners controlling for dyads' age, gender, education level and self‐efficacy respectively.

**Result:**

The sample consisted of 82 dyads comprising patients with MCI (59.8% male, average age 72.6 years, average education 16.5 years) and their care partners (24.4% male, average age 66.8 years, average education 15.5 years). The average self‐efficacy, depression and anxiety were 203.6±28.7, 6.51±4.29 and 26.0±6.01 for patient and 202.4±36.9, 7.61±7.66 and 26.8±7.40 for care partner respectively. Regarding depression, there was a significant actor effect of self‐efficacy for both patients and care partners. Increased self‐efficacy was associated with decreased depression for both patients (β = ‐0.07, P<0.001) and care partners (β = ‐0.11, P<0.001). A marginally significant partner effect of care partner’s self‐efficacy on patient depression was observed (β = ‐0.05, P = 0.066), but no effect of patient’s self‐efficacy on caregiver’s depression was found. For anxiety, there was a significant actor effect of self‐efficacy for both patients and care partners. Increased self‐efficacy was associated with decreased anxiety for both patients (β = ‐0.11, P<0.001) and care partners (β = ‐0.11, P<0.001). However, no partner effects were observed.

**Conclusion:**

Our findings emphasize the importance of considering patient/care partner dyads to better understand the complex interplay between psychological outcomes and predictors in the context of mild cognitive impairment.

